# The role of a labial salivary gland biopsy in the diagnostic procedure for Sjögren’s syndrome; a study of 94 cases

**DOI:** 10.4317/medoral.20010

**Published:** 2014-06-01

**Authors:** Dewi van Stein-Callenfels, Jonathan Tan, Elisabeth Bloemena, Richard M. van Vugt, Alexandre E. Voskuyl, Nathalie T.Y. Santana, Isaäc van der Waal

**Affiliations:** 1 VU University Medical Center, Department of Oral and MaxilloFacial Surgery/ Oral Pathology and Academic Centre of Dentistry Amsterdam ACTA, Amsterdam, the Netherlands; 2 VU University Medical Center, Department of Pathology, the Netherlands; 3 VU University Medical Center, Department of Rheumatology, the Netherlands; 4 VU University Medical Center, Department of Ophthalmology, the Netherlands

## Abstract

Objectives: The purpose of the present study is to examine the role of the outcome of the labial salivary gland biopsy (LSGB) in the diagnostic procedure of patients suspected of suffering from Sjögren’s syndrome (SS). Material and Methods: In a retrospective study the result of histopathological assessment of 94 consecutively taken labial salivary gland biopsies has been examined. For the diagnosis of SS the American-European Consensus Group classification (AECG, 2002) have been used. The outcome of the assessment has been discussed in relation to a recently reported classification provided by the American College of Rheumatology (ACR, 2012). Results: In the 94 LSGBs support for a diagnosis of SS has been encountered in 24 out of 26 patients with SS. In the 68 patients with a negative diagnosis of SS only six positive LSGBs were observed. The sensitivity of the labial biopsy amounted 0.92; the specificity was 0.91, while the positive predictive value and the negative predictive value amounted 0.80 and 0.97 respectively. LSGBs taken by or on the request of the departments of Rheumatology or Internal Medicine had a significant higher yield compared to LSGBs taken in other clinical departments. Conclusions: The LSGB may play a role in the diagnostic procedure of Sjögren’s syndrome when using either the AECG classification or the ACR classification. A LSGB should preferably taken after counseling for the possible presence of SS by a department of Rheumatology or Internal Medicine since the yield of such biopsies is much higher than in patients who have not been counseled by these departments prior to the taking of a LSGB. When using the ACR classification, a positive serologic result and a positive ocular test make the taking of a LSGB redundant. Only in case of a negative serologic outcome or a negative result of the ocular test a LSGB is indicated. Since both the serologic test and the ocular test carry hardly any morbidity, these tests should, indeed, be performed first before considering to take a LSGB.

** Key words:**Labial salivary gland biopsy, Sjögren’s syndrome.

## Introduction

Sjögren’s syndrome (SS) is a multiorgan, chronic autoimmune disease, primarily directed against exocrine glands, being characterized by dry mouth and dry eyes. Histopathologically, clusters of monocellular immune cells are present in the exocrine glandular tissues, leading to atrophy. In SS fatigue, arthritis and kidney failure may occur and, as a late complication, the development of non-Hodgkin lymphoma. However, the majority of these ‘signature’ symptoms may also be related to other diseases. SS may have a genetic predilection, as well as hormonal and environmental etiological factors. All of this adds to the complexity of the disease (1,2).

SS may exist as a single condition, being called primary Sjögren’s syndrome (pSS). Sjögren’s syndrome can also be associated with other autoimmune diseases, being referred to as secondary Sjögren’s syndrome (sSS). The prevalence of SS is estimated to be 0,5-1% of the population; there is a distinct female preponderance (3).

In the past decades several diagnostic criteria have been proposed to facilitate the diagnosis of SS. The most commonly used classification is the one proposed by the American-European Consensus Group (AECG) (1). Of the six diagnostic criteria in this classification at least four should be positive in order to qualify for a diagnosis of SS. The histopathological findings in a biopsy of the labial salivary glands, usually taken from the lower lip, is one of the six criteria. A positive labial salivary gland biopsy (LSGB) should have a focus score of more than one. A focus is defined as the presence of a cluster of at least 50 lymphocytes per 4 mm2 glandular tissue adjacent to normal appearing mucous acini.

The purpose of the present study is to examine the role of the outcome of the LSGB in the 

diagnostic procedure of patients suspected of suffering from SS.

## Material and Methods

In the files of the department of pathology of the VU university medical center, Amsterdam, the Netherlands, 139 consecutive labial salivary gland biopsies (LSGBs) could be retrieved in the period between 2000 and 2010. Demographic, clinical and laboratory data from these patients were obtained from the medical records. Out of these 139 cases, 45 patients have been excluded because of incomplete data. As a result 94 patients have been included in the study. There were 74 females and 20 males; the mean age at the time of biopsy was 50 years (range 21 to 79 years). For the diagnosis of SS the AECG criteria have been used (4). The histopathological assessment of the presence of lymphocytic foci in the labial salivary gland biopsies has been performed in a quantitative way (5). For the statistical analyses SPSS 21.0 for Windows was used, calculating the Pierson’s chi-squared test.

The design of this study adheres to the code for proper secondary use of human tissue of the Dutch Federation of Biomedical Scientific Societies (http://www.federa.org).

## Results

The overall results of the various diagnostic tests are listed in  Table 1. Of the 26 SS positive patients the mean age is 47 years.

**Table 1:**
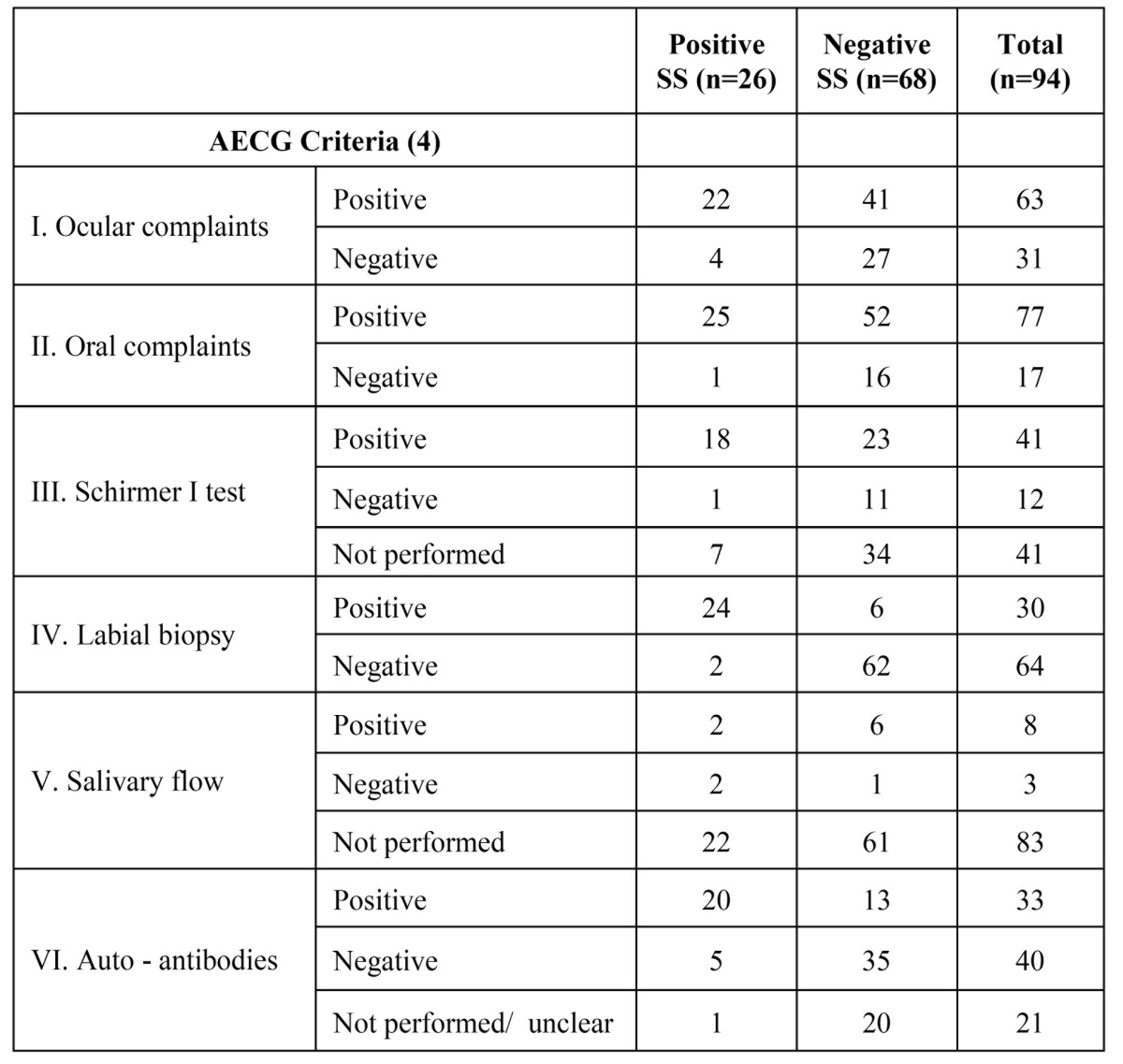
Results of the various diagnostic tests in 94 patients in whom a labial salivary gland biopsy has been performed.

Of the 64 negative labial biopsies four did not contain sufficient glandular tissue to allow a focus score. The sensitivity of the labial biopsy amounted 0.92; the specificity was 0.91, while the positive predictive value and the negative predictive value amounted 0.80 and 0.97 respectively.

In  Table 2 the results of the various diagnostic tests in the patients with a final diagnosis of SS, divided in primary (n=20) and secondary (n=2) SS are shown. In four patients no reliable data could be retrieved from the clinical records to allow a distinction between primary and secondary SS. Apparently, the results of the salivary flow test have not been recorded or this test has not been performed in all but a few patients.

**Table 2:**
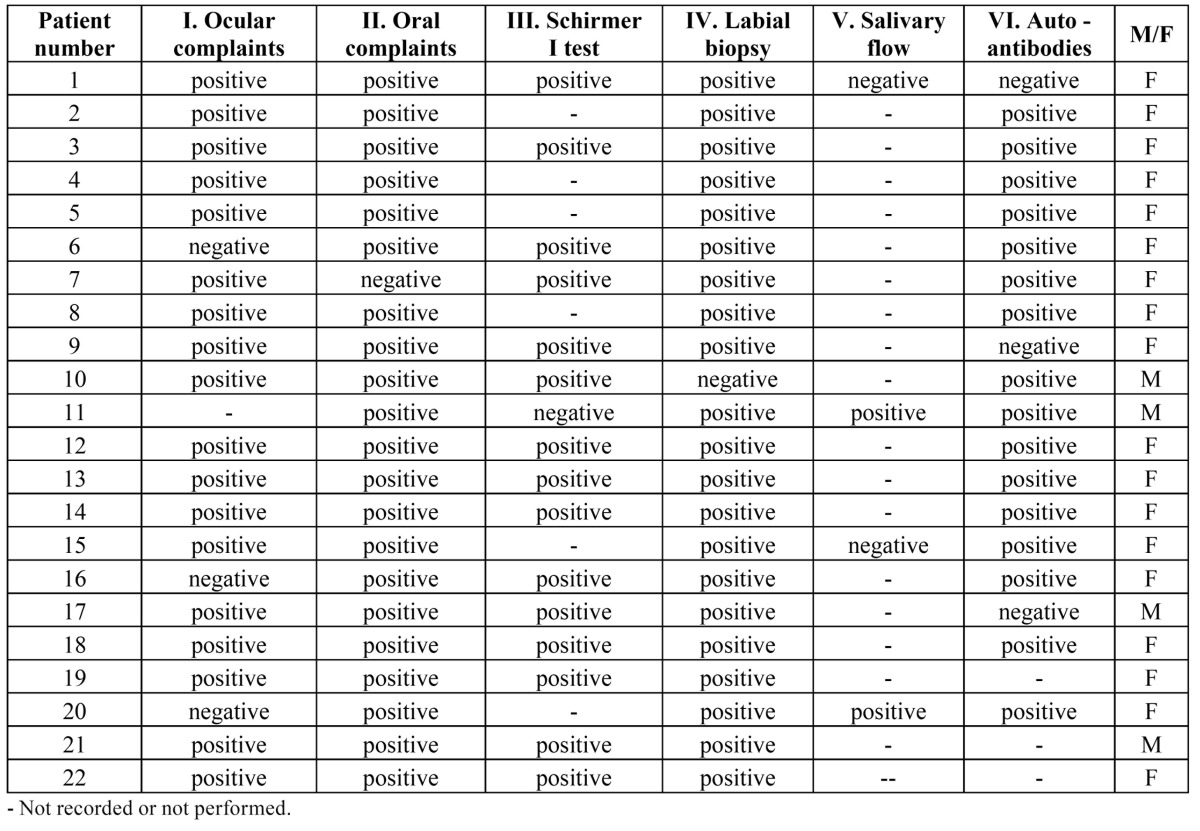
The results of the various diagnostic tests in 20 patients with primary SS (#1-20) and two patients with secondary SS (#21 and 22).

The percentages of positive labial biopsies for each department are shown in  Table 3. The percentage of positive LSGBs was significantly higher in patients in whom the biopsy was performed by or on the request of either the department of Rheumatology and Internal Medicine compared to patients who had not been counseled by these departments prior to the taking of the LSGB (*p* < 0,05).

**Table 3:**
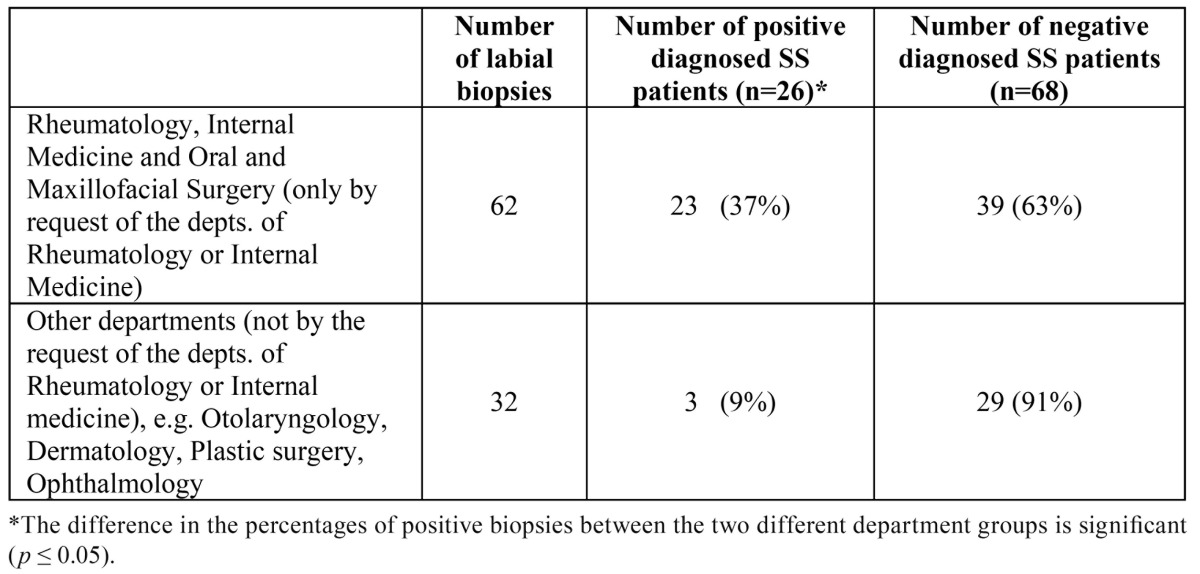
Result of labial salivary gland biopsy per department groups.

The comparison of the result of the labial biopsy and the serology obtained in the 20 patients diagnosed with primary Sjögren syndrome shows that in case of a positive LSGB (n=19) the serology was positive in 80% per cent of the cases, while in patients with a positive serology (n=16) only one negative LSGB was encountered. In case of a negative result of the serology (n=4) a positive LSGB was observed in three patients.

## Discussion and Conclusions

The present study has been undertaken for two reasons. Firstly, we wanted to examine in what way labial biopsies taken in our Institution in a 10-year period had contributed to the assessment of a diagnosis of Sjögren’s syndrome. Secondly, we wanted to explore the influence of the recently proposed ACR criteria with regard to the role of the LSGB.

Of the 94 LSGBs that were suitable for evaluation only 26 have contributed to a final diagnosis of SS. As can be seen from the figures in Table 1 the Schirmer I test has been performed in approximately half of the patients only. Measurement of the salivary flow has been performed in only 12 percent of the patients. This is remarkably since a LSGB carries some degree of morbidity; some two per cent of patients experience long term postoperative complaints of numbness or hyperaesthesia at the site of the biopsy (6).

As is shown in  Table 3, the yield of LSGBs has been significant higher in patients in whom the biopsy was performed by or by the request from the departments of Rheumatology or the department of Internal Medicine than in patients in whom the biopsies were taken in other departments, not by the request from the departments of Rheumatology or Internal Medicine. Therefore, it seems advisable to perform LSGBs only after counseling of the patients by the departments of Rheumatology or Internal Medicine.

Our results show a high specificity and a high sensitivi-ty of the LSGB with regard to the presence of SS ( Table 1). These results are somewhat similar to the outcome of a study by Baeteman e.a., who calculated an even higher specificity of 1,00 and a sensitivity of 0,75 (7). It is well recognized that the assessment of a focus score, when performed in a semiquantitive fashion, carries room for discussion about its value (8,9). In postmortem studies older age was associated with high false-positive rates of LSGB (10). Iin view of a mean age of 50 years at the time of the biopsy, age does not seem to have a major influence on the present results.

In the past, it has been suggested that immunohistochemical assessment of the various percentages of immunoglobulins in the plasma cells, such as IgA, IgG and IgM, has a prognostic significance with regard to future development of malignant lymphoreticular disease (11); the same research group has shown that quantitative immunohistologic criteria are superior to the lymphocytic focus score for the diagnosis of SS (12). However, no other studies on these two subjects have been published and they have not been incorporated in the AECG classification nor in the ACR classification.

In 2012 a revised classification has been proposed by the American College of Rheumatology (ACR) in which the subjective criteria of dry mouth and dry eyes have been deleted, focusing on 1) serologic findings, 2) a focus score in labial salivary gland biopsies, and 3) ocular signs (13). Furthermore, no distinction is made in this classification between primary and secondary SS. For the assessment of the ocular signs in the ACR classification ocular surface staining has been performed instead of a Schirmer test. In a study comparing the two classifications (AECG versus ACR) no significant difference was found between the diagnostic value of these two classifications (14). When using the ACR classification, a positive serologic result and a positive ocular test make the taking of a LSGB redundant (15,16). Only in case of a negative serologic outcome or a negative result of the ocular test a LSGB is indicated. Since both the serologic test and the ocular test carry hardly any morbidity, these tests should be performed first before considering to take a LSGB.

## References

[1] Baldini C, Talarico R, Tzioufas AG, Bombardieri S (2012). Classification criteria for Sjögren's syndrome: a critical review. J Autoimmun.

[2] Bowman SJ, Fox RI (2014). Classification criteria for Sjögren's syndrome: nothing ever stands still. Ann Rheum Dis.

[3] Guellec D, Cornec D, Jousse-Joulin S, Marhadour T, Marcorelles P, Pers JO (2013). Diagnostic value of labial minor salivary gland biopsy for Sjögren's syndrome: a systematic review. Autoimmun Rev.

[4] Vitali C, Bombardieri S, Jonsson R, Moutsopoulos HM, Alexander EL, Carsons SE (2002). Classification criteria for Sjögren's syndrome: a revised version of the European criteria proposed by the American-European Consensus Group. Ann Rheum Dis.

[5] de Wilde PC, Baak JP, Slootweg PJ, Hené RJ, Kater L (1986). Morphometry in the diagnosis of Sjögren's syndrome. Anal Quant Cytol Histol.

[6] Richards A, Mutlu S, Scully C, Maddison P (1992). Complications associated with labial salivary gland biopsy in the investigation of connective tissue disorders. Ann Rheum Dis.

[7] Baeteman C, Guyot L, Bouvenot J, Chossegros C, Cheynet F, Loudot C (2008). Faut-il encore effectuer des biopsies des glandes salivaires accessoires?. Rev Stomatol Chir Maxillofac.

[8] Vivino FB, Gala I, Hermann GA (2002). Change in final diagnosis on second evaluation of labial minor salivary gland biopsies. J Rheumatol.

[9] Stewart CM, Bhattacharyya I, Berg K, Cohen DM, Orlando C, Drew P (2008). Labial salivary gland biopsies in Sjögren's syndrome: still the gold standard?. Oral Surg Oral Med Oral Pathol Oral Radiol Endod.

[10] Takeda Y, Komori A (1986). Focal lymphocytic infiltration in the human labial salivary glands: a postmortem study. J Oral Pathol.

[11] Bodeutsch C, de Wilde PC, Kater L, van den Hoogen FH, Kruize AA, Ebben GP (1992). Quantitative immunohistologic study of lip biopsies. Evaluation of diagnostic and prognostic value in Sjögren's syndrome. Pathol Res Pract.

[12] Bodeutsch C, de Wilde PC, Kater L, van Houwelingen JC, van den Hoogen FH, Kruize AA (1992). Quantitative immunohistologic criteria are superior to the lymphocytic focus score criteria for the diagnosis of Sjögren's syndrome. Arthritis Rheum.

[13] Shiboski SC, Shiboski CH, Criswell L, Baer A, Challacombe S, Lanfranchi H (2012). American College of Rheumatology classification criteria for Sjögren's syndrome: a data-driven, expert consensus approach in the Sjögren's International Collaborative Clinical Alliance cohort. Arthritis Care Res (Hoboken).

[14] Rasmussen A, Ice JA, Li H, Grundahl K, Kelly JA, Radfar L (2014). Comparison of the American-European Consensus Group Sjögren's syndrome classification criteria to newly proposed American College of Rheumatology criteria in a large, carefully characterised sicca cohort. Ann Rheum Dis.

[15] Manthorpe R (2002). Sjögren's syndrome criteria. Ann Rheum Dis.

[16] Bamba R, Sweiss NJ, Langerman AJ, Taxy JB, Blair EA (2009). The minor salivary gland biopsy as a diagnostic tool for Sjogren syndrome. Laryngoscope.

